# Entropy-Augmented Forecasting and Portfolio Construction at the Industry-Group Level: A Causal Machine-Learning Approach Using Gradient-Boosted Decision Trees

**DOI:** 10.3390/e28010108

**Published:** 2026-01-16

**Authors:** Gil Cohen, Avishay Aiche, Ron Eichel

**Affiliations:** Management Department, Western Galilee College, Acre 2412101, Israel; avishaya@wgalil.ac.il (A.A.); rone@wgalil.ac.il (R.E.)

**Keywords:** portfolio, machine learning, gradient boosting, industry

## Abstract

This paper examines whether information-theoretic complexity measures enhance industry-group return forecasting and portfolio construction within a machine-learning framework. Using daily data for 25 U.S. GICS industry groups spanning more than three decades, we augment gradient-boosted decision tree models with Shannon entropy and fuzzy entropy computed from recent return dynamics. Models are estimated at weekly, monthly, and quarterly horizons using a strictly causal rolling-window design and translated into two economically interpretable allocation rules, a maximum-profit strategy and a minimum-risk strategy. Results show that the top performing strategy, the weekly maximum-profit model augmented with Shannon entropy, achieves an accumulated return exceeding 30,000%, substantially outperforming both the baseline model and the fuzzy-entropy variant. On monthly and quarterly horizons, entropy and fuzzy entropy generate smaller but robust improvements by maintaining lower volatility and better downside protection. Industry allocations display stable and economically interpretable patterns, profit-oriented strategies concentrate primarily in cyclical and growth-sensitive industries such as semiconductors, automobiles, technology hardware, banks, and energy, while minimum-risk strategies consistently favor defensive industries including utilities, food, beverage and tobacco, real estate, and consumer staples. Overall, the results demonstrate that entropy-based complexity measures improve both economic performance and interpretability, yielding industry-rotation strategies that are simultaneously more profitable, more stable, and more transparent.

## 1. Introduction

Recent progress in quantitative finance highlights the growing importance of information-theoretical complexity as a complementary dimension to traditional predictive modeling. While classical approaches rely mainly on momentum, volatility, and cross-sectional dispersion, measures such as Shannon entropy and fuzzy entropy capture irregularity and temporal instability in return dynamics that conventional indicators often overlook. This study examines how complexity-based features can improve the accuracy and robustness of industry-group-level return forecasting and portfolio construction within a strictly causal framework built on gradient-boosted decision trees (XGBoost). The empirical design covers multiple investment horizons, including weekly, monthly, and quarterly intervals. All analysis is performed at the industry level, where systematic drivers dominate idiosyncratic noise. Industry indices provide an effective testing ground for regime-sensitive modeling because they reflect macroeconomic conditions, policy shifts, and investor sentiment across industrial domains such as technology, energy, healthcare, and financials. By integrating entropy-based features into this cross-industry environment, we evaluate whether measures of local irregularity and return dispersion can enhance predictive power and improve tactical and strategic allocation outcomes.

Industry allocation has long been recognized as a central dimension of portfolio design because industry groups differ in their exposure to macroeconomic drivers such as interest rates, inflation, and economic growth. For instance, technology-related industries typically behave like long-duration assets, becoming more sensitive to increases in discount rates, while commodity-linked industries such as Energy and Materials tend to outperform during inflationary or supply-driven shocks. Financial industries often benefit from moderate rate hikes, whereas cost-sensitive industries such as Consumer Discretionary and Industrials may experience margin compression when input costs rise. These contrasting sensitivities create natural hedging dynamics across industry groups. Consequently, a balanced allocation across GICS Industry Groups can stabilize portfolio volatility and improve performance resilience across changing macroeconomic regimes.

The modeling approach is strictly data driven. For each horizon, we estimate forward returns using rolling-window XGBoost regressors and classifiers trained on a rich feature set that includes momentum, trend deviation, volatility, cross-sectional ranks, and two complexity indicators. Shannon entropy measures the dispersion of recent return distributions, while fuzzy entropy captures temporal irregularity using delay-vector similarity decay. These features act as regime detectors that adjust model confidence based on the stability of recent market behavior. The resulting hybrid models produce directional probabilities and expected-return magnitudes, which are combined through adaptive weighting that reflects out-of-sample performance.

We use the predictive outputs to construct two types of portfolios. The maximum-profit strategy allocates more weight to industries with stronger combined predictive signals, while the minimum-risk strategy uses a dynamic covariance matrix to identify risk-efficient allocations. Both portfolios are implemented as non-overlapping horizon-based strategies, ensuring causal validity and independent evaluation over more than three decades of data.

The empirical results show consistent performance improvements from incorporating entropy features. Shannon and fuzzy entropy raise average annual returns by up to two percentage points in short-horizon strategies and reduce volatility in risk-controlled portfolios. These enhancements persist across market regimes, confirming that complexity-aware modeling improves both profitability and stability by embedding regime sensitivity directly into the predictive process.

This research contributes to literature in three main ways. First, it provides large-sample evidence that information-theoretic complexity adds measurable predictive value in industry-level forecasting. Second, it presents a transparent and interpretable ensemble framework that integrates entropy-based regime awareness into a modern machine-learning structure. Third, it links forecasting to portfolio construction, showing that complexity-informed predictors support both tactical responsiveness and long-term stability. As financial markets become more volatile and structurally fragmented, models that incorporate complexity awareness will be increasingly essential for adaptive portfolio management. The findings in this paper establish entropy-augmented modeling as a robust foundation for the next generation of data-driven investment strategies.

## 2. Literature Review

The integration of artificial intelligence into financial forecasting has transformed the analysis of return dynamics at the industry and industry-group levels. Traditional econometric tools often struggle with nonlinearity, structural breaks, and cross-industry interactions. As a result, recent work increasingly employs machine learning, entropy-based complexity measures, and hybrid modeling schemes that combine diverse sources of information. This section reviews four relevant research domains: machine learning in technical analysis, industry analysis, entropy and fuzzy modeling in financial time series and hybrid frameworks that integrate heterogeneous predictors.

### 2.1. Machine Learning in the Technical Analysis of Industry Group Indices

Early empirical work, such as Neely, Weller, and Dittmar [1] and Dawson and Steeley [2], demonstrated that systematic technical rules can extract informative patterns from price series. The transition to machine learning significantly expanded these ideas. Souza et al. [3] showed that algorithmic enhancements to moving-average rules improve profitability across major emerging markets. Shi and Zhao [4] demonstrated that deep neural networks adapt more effectively than static rules to evolving market conditions, particularly when regimes shift or volatility spikes. Dey [5] provided a broad overview emphasizing the suitability of ML for nonlinear environments, and Hurriyati et al. [6] documented the improvement in trend classification achieved through ML-based predictive evaluation.

While early studies focused on individual assets, more recent research addresses aggregated index structures. Sonani, Badii, and Moin [7] combined LSTM and graph-neural-network components to capture both temporal and cross-sectional dependencies in index-level returns. These works collectively underscore the growing role of ML in refining technical signals and in identifying dynamic relationships across industry groups.

While this literature demonstrates the effectiveness of machine learning methods in capturing nonlinear dynamics and cross industry interactions, it largely relies on price based, technical, or fundamental predictors. Measures of market complexity derived from information theory are rarely incorporated directly into these forecasting models, particularly in an industry level setting.

### 2.2. Industry-Based Return Predictability, Machine Learning Asset Pricing, and Information Theoretic Foundations

Research on industry level return predictability shows that industries linkages, information diffusion, and structural differences across industries play a central role in shaping expected returns. Cohen and Frazzini [8] demonstrated that economically connected industries tend to exhibit predictable return spillovers because information travels through production and supplier networks. This finding highlights that industry relationships contain meaningful structure that can be used for forecasting. Kacperczyk, Van Nieuwerburgh, and Veldkamp [9] further showed that industries differ in the informativeness of firm level disclosures. Their results indicate that predictability at the industry level depends not only on systematic risk exposures but also on variation in information precision and attention across industries. Hou, Mo, and Zhang [10] advanced this perspective by developing the q factor model. Their framework emphasizes investment and profitability based factors that differ across industries and provides a modern structural interpretation of return predictability.

In parallel, machine learning asset pricing literature has developed methods that directly inform the forecasting design of the present study. Gu, Kelly, and Xiu [11] showed that nonlinear machine learning models such as tree based, and neural network architectures outperform linear models in predicting cross sectional returns. Their findings demonstrate that machine learning methods can capture complex interactions among technical indicators, fundamentals, and macroeconomic variables. In a follow up study, Gu, Kelly, and Xiu [12] showed that these models remain stable across changing market conditions when appropriate regularization and out of sample validation are applied. Bryzgalova, Julliard, and Zhu [13] provided additional empirical support by examining high dimensional forecasting. They showed that models with flexible structure and appropriate shrinkage are particularly effective in extracting persistent cross sectional patterns. This literature forms a methodological foundation for applying machine learning techniques to industry level forecasting in a strictly out of sample and horizon dependent setting.

A third foundational component relates to information theory and the measurement of complexity in financial time series. Shannon [14] introduced the concept of entropy as a general measure of uncertainty in probabilistic systems. This framework provides the theoretical basis for quantifying the dispersion and unpredictability of return distributions. Building on this foundation, Zunino et al. [15] applied entropy-based methods to financial markets and showed that entropy captures the degree of informational efficiency. Baruník and Křehlík [16] further demonstrated that information theoretic measures reflect evolving market complexity and nonlinear dependence structures introduced a frequency domain framework for measuring time varying connectedness across financial markets. Their results show that market linkages differ across short- and long-term investment horizons, and that systemic risk propagates through distinct frequency channels. This perspective complements entropy-based measures by highlighting that market complexity arises not only from local irregularity but also from horizon dependent interconnections among industries. Incorporating these insights strengthens the theoretical motivation for using information theoretical features in multi horizon forecasting models. These insights support the use of both Shannon entropy and fuzzy entropy as regime sensitive indicators within machine learning forecasting frameworks. Industry indices often display heterogeneous volatility, structural breaks, and variation in return dispersion. Information theoretical features are therefore well suited to characterize such dynamics in a predictive and adaptive manner.

Most studies in this stream employ entropy as a descriptive or diagnostic tool, using it to assess market efficiency, informational disorder, or evolving dependence structures. In these applications, entropy is typically computed ex post and interpreted as a characterization of observed market behavior rather than as an explanatory variable that actively conditions forecasts.

As a result, despite the rich theoretical foundations of information theory in finance, there remains limited empirical work that integrates entropy measures directly into predictive models of returns. In particular, the use of entropy as a regime-sensitive feature within causal forecasting frameworks at the industry group level has received little systematic attention.

### 2.3. Entropy-Based Methods in Financial Markets

Entropy-based measures are widely used in the analysis of financial markets as information-theoretic tools for characterizing complexity, nonlinear dependence, and deviations from randomness in economic time series. Zunino et al. [17] demonstrated that permutation entropy provides a robust framework for identifying forbidden patterns and structural inefficiencies in asset returns, capturing dynamical properties that are not accessible through second-moment statistics or linear models. Building on this line of research, Bariviera [18] applied entropy-based measures to digital asset markets and documented time-varying informational efficiency and regime-dependent dynamics in environments characterized by rapid structural change.

A parallel strand of the literature employs information-theoretic approaches to analyze directional information flows and lead–lag relationships across financial assets. Fiedor [19] showed that entropy-based measures are particularly effective in uncovering asymmetric and nonlinear interactions in multivariate financial systems. Extending this perspective, Diebold and Yilmaz [20] integrated entropy-related concepts into the analysis of volatility spillovers and financial connectedness, demonstrating how information-based measures complement network and spillover frameworks, especially under conditions of elevated uncertainty and systemic stress. More recently, Pinto et al. [21] confirmed the relevance of entropy-based indicators for assessing market efficiency during crisis periods, showing systematic shifts in entropy measures across tranquil and turbulent regimes.

Within this established and methodologically diverse literature, the present study contributes a focused empirical application of an entropy-based measure in a specific financial context. Rather than introducing entropy methods per se, the analysis aims to provide additional evidence on their usefulness for characterizing market dynamics and informational structure under well-defined institutional conditions.

### 2.4. Entropy and Fuzzy Logic

Entropy-based approaches provide tools for quantifying uncertainty, irregularity, and local complexity in financial data. Shannon entropy captures the dispersion of return distributions, while fuzzy entropy evaluates the structural irregularity of embedded vectors in short time windows. Wu and Zhang [22] applied fuzzy entropy to international indices and highlighted its ability to identify volatility patterns and structural instability. Zhou et al. [23] integrated entropy constraints into portfolio selection, showing improved robustness relative to classical mean-variance optimization. Yang, Yu, and Ralescu [24] applied entropy-augmented fuzzy time-series models to portfolio optimization, and Alizadeh et al. [25] developed an adaptive neuro-fuzzy system that accounts for nonlinear relationships in asset behavior.

Shannon entropy and fuzzy entropy capture distinct dimensions of market complexity. Shannon entropy reflects the dispersion of return distributions and is therefore sensitive to changes in uncertainty and probabilistic disorder. Fuzzy entropy, in contrast, is designed to quantify temporal irregularity and structural instability by evaluating similarity decay in embedded return sequences. Prior studies applying fuzzy entropy emphasize its ability to detect regime shifts and nonlinear dynamics, particularly in environments where volatility-based measures provide an incomplete description of instability. These differences motivate the joint use of both measures within forecasting systems.

The present study extends these lines of research by embedding Shannon and fuzzy entropy directly into a causal ML forecasting system that operates across weekly, monthly, and quarterly investment horizons. Instead of applying entropy solely to volatility modeling or optimization constraints, the complexity measures here serve as predictive features that inform model confidence, regime characterization, and cross-industry differentiation.

### 2.5. Hybrid Machine Learning Models and Adaptive Weighting

A recent trend in financial forecasting involves hybrid ML systems that combine heterogeneous signal types within unified predictive frameworks. Singh [26] and Zhao [27] showed that models integrating technical and fundamental indicators improve forecast robustness. Abraham et al. [28] combined genetic algorithms with random forests to enhance directional prediction accuracy, while Ramesh et al. [29] examined robo-advisory systems that rebalance portfolios using ML-driven decision rules. Ayyildiz and Iskenderoglu [30] compared multiple ML architectures for index prediction and concluded that ensemble-based systems offer the strongest and most stable performance.

Cohen, Aiche, and Eichel [31] proposed a structured framework that integrates semantic signals, information-theoretic features, and traditional predictors. Their findings are consistent with the present study, which embeds entropy-based complexity measures within gradient-boosted decision trees and combines directional and magnitude forecasts through adaptive weighting across rolling horizons.

The current paper contributes to this literature by demonstrating the effectiveness of integrating entropy-based complexity indicators into a gradient-boosted ensemble framework. By combining diverse signal types with adaptive weighting mechanisms, the approach yields improved predictive accuracy and more resilient portfolio construction. The findings support the development of modular, flexible forecasting systems that adjust input weights in real time according to model performance, narrative influence, and regime context. This contributes theoretically and practically to the understanding of how complexity-aware features interact with traditional modeling techniques at the industry-group level.

Overall, the existing literature highlights the value of entropy for describing market complexity and for improving portfolio robustness, yet it rarely embeds entropy directly within causal machine learning forecasting architectures. Moreover, few studies examine entropy-based predictors at the industry group level across multiple investment horizons. This paper contributes to the literature by integrating Shannon and fuzzy entropy as internal features within a rolling window machine learning framework and by linking entropy conditioned forecasts to economically interpretable portfolio construction rules.

## 3. Data and Feature Engineering

The empirical analysis is based on daily time-series data for 25 U.S. equity indices classified under the Global Industry Classification Standard (GICS) at the Industry Group level. The dataset spans the period from 2 January 1992 to 31 December 2024, providing a consistent and comprehensive time series that captures multiple market cycles, including periods of expansion, contraction, and structural regime shifts. All indices were retrieved from standardized financial databases and cross-validated with publicly available benchmark data to ensure continuity and accuracy. The use of GICS Industry Groups offers a balanced level of granularity that reflects industrial rotation dynamics while preserving statistical coherence across the full three-decade horizon. Within the GICS taxonomy, the Industry Group level provides an intermediate granularity between broad sectors (11 categories) and specific industries (74 categories). This level balances interpretability with statistical stability, making it ideal for studying industry rotation and cross-industry dynamics.

Let $P_{s,t}$ denote the closing index value for industry s on trading day t. The dataset is transformed into a long-panel structure with observations indexed by industry and date, which allows both time-series computations within industries and cross-sectional computations across industries. The fundamental input variable for forecasting is the simple daily return

(1) $$r_{s,t}=\frac{P_{s,t}}{P_{s,t-1}}-1,$$

computed without forward-filling and used consistently throughout the feature set and model training pipeline.

A broad collection of time-series technical indicators is constructed for each industry using only lagged observations, ensuring strict causality and eliminating look-ahead bias. Momentum measures are created over windows of one, five, twenty-one, and sixty-three days by computing rolling sums of past returns. These indicators capture short-term, medium-term, and long-term directional trends in industry performance. Measures of return variability are obtained through twenty-one-day and sixty-three-day rolling standard deviations, providing estimates of local volatility. In addition, twenty-one-day rolling maximum and minimum of returns serve to capture the presence of recent extremes, such as industry-specific drawdowns or abrupt recoveries.

To characterize deviations from medium-term price trends, moving-average ratios are constructed for windows of ten, twenty-one, and fifty days. Each ratio takes the form $(P_{s,t}/{\mathrm{MA}}_{s,t}(m))-1$, where the moving average is computed using only information available up to day t−1. These indicators measure whether the industry is trading above or below its smooth trend level. Oscillator-type indicators are also included. The relative strength index (RSI) is computed using a fourteen-day window of gains and losses and lagged by one day. The moving-average convergence–divergence (MACD) indicator and its nine-day signal line are constructed through differences in exponential moving averages with spans of twelve and twenty-six days, also lagged appropriately. Bollinger-band statistics are implemented through a twenty-day moving average and rolling standard deviation, yielding both a normalized z-score and the relative band width. A stochastic %K indicator, computed as the normalized position of the current price within its recent fourteen-day high–low range, provides an additional measure of local overbought or oversold conditions.

Beyond technical indicators, the feature set includes two information-theoretic complexity measures computed from short windows of recent returns. Shannon entropy is estimated to be using a twenty-day rolling window. The empirical distribution of returns in the window is discretized into histogram bins, and the entropy

(2) $$H_{s,t}=-\sum_{k}p_{s,t,k}\;log\;p_{s,t,k}$$

is evaluated using only nonzero bin probabilities. This quantity increases when the distribution of recent returns is diffuse, reflecting heightened uncertainty or regime instability. A fuzzy-entropy measure is computed using delay-vector embeddings of dimension two. Within each window, pairs of embedded vectors are compared through an exponential similarity kernel of the form exp(−d/r), where d is the maximum coordinate difference and r is a scale parameter proportional to the local volatility. The entropy estimate is based on the decay of average similarity when the embedding dimension increases. This provides a continuous, non-discretized measure of temporal irregularity in industry return dynamics.

The feature engineering process also incorporates cross-sectional information. On each trading day, the vector of industry returns is standardized to produce a cross-sectional z-score, and a corresponding cross-sectional rank between zero and one. The same z-score and rank transformations are applied to the twenty-one-day momentum measure. These features capture the relative positioning of each industry within the broader market at time t, enabling the model to learn patterns of industry rotation and cross-industry dispersion.

All features are lagged by one day where required, ensuring that every predictor observable at time t uses only information available strictly prior to the prediction date. The resulting feature matrix combines time-series technical indicators, entropy-based complexity measures, and cross-sectional descriptors of relative performance. This rich, strictly causal feature set serves as the input to the gradient-boosted decision tree models described in the methodology section and forms the empirical foundation for the construction of maximum-profit and minimum-risk portfolios across weekly, monthly, and quarterly investment horizons.

**Figure 1:**
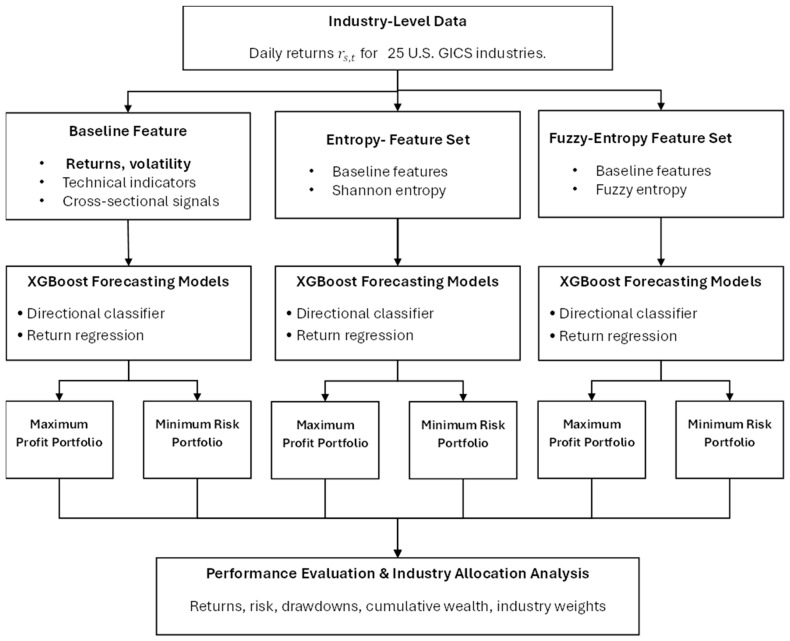
Entropy-augmented XGBoost framework for industry-level forecasting and portfolio construction.

To clarify the overall structure of the forecasting and allocation framework, Figure 1 provides a schematic overview of the parallel model pipelines employed in the empirical analysis. The figure illustrates how identical industry-level input data are transformed into three distinct feature representations, each of which feeds an independent XGBoost forecasting model that produces both maximum-profit and minimum-risk portfolios. This visual summary complements the formal model description by highlighting the strictly causal, parallel design of the entropy-augmented architecture.

## 4. Model Architecture and Portfolio Construction

The modeling framework combines gradient-boosted decision trees and information-theoretically complexity measures to forecast industry-level returns across multiple investment horizons and convert these forecasts into economically meaningful portfolio decisions. Let $P_{s,t}$ denote the closing index value for industry s on trading day t. Consistent with the empirical construction described above, the fundamental time-series input to the forecasting models is the simple daily return

(3) $$r_{s,t}=\frac{P_{s,t}}{P_{s,t-1}}-1.$$

which forms the fundamental time-series input for all subsequent modeling stages. For each horizon H∈{5,21,63}, corresponding approximately to weekly, monthly, and quarterly holding periods, the realized forward return is

(4) $$R_{s,t}^{\left(H\right)}=\frac{P_{s,t+H}}{P_{s,t}}-1.$$

This continuous quantity forms the target for the regression models. The corresponding directional variable is

(5) $$Y_{s,t}^{\left(H\right)}=\left\{\begin{array}{cc} 1, & \;\mathrm{if}\;R_{s,t}^{\left(H\right)}>0,\\ 0, & \mathrm{otherwise}, \end{array}\right.$$

which is used for classification models. All targets are constructed using information available at time t, so the predictive tasks are strictly causal.

The primary predictive engine consists of gradient-boosted decision trees implemented via XGBoost. For each horizon H, two separate models are estimated in rolling windows aligned with the forecasting horizon. The classification model approximates the conditional probability of a positive forward return,

(6) $${\hat{p}}_{s,t}^{\left(H\right)}\approx \mathrm{Pr}\;\left(Y_{s,t}^{\left(H\right)}=1|F_{t}\right),$$

where $F_{t}$ denotes the sigma-algebra generated by all engineered technical, cross-sectional, entropy, and fuzzy-entropy features up to time t. The regression model estimates the conditional expectation of the forward return,

(7) $${\hat{R}}_{s,t}^{\left(H\right)}\approx E[R_{s,t}^{\left(H\right)}|F_{t}].$$

Both models are trained on a rolling window of historical observations whose length is chosen to be proportional to the investment horizon, so that short-horizon predictions emphasize recent information, while longer horizons draw on a broader history. Each rolling window is partitioned into a training subset and an internal validation subset. The validation subset is used to compute accuracy, precision, recall, F-score, area under the ROC curve, and mean-squared prediction error, and to determine the optimal directional classification threshold $\tau_{H}$ that maximizes the out of sample F-score. This adaptive threshold selection ensures that the classifier responds flexibly to evolving return distributions, volatility regimes, and cross-industry dynamics.

Beyond these predictive models, the architecture incorporates two information-theoretic measures constructed from recent returns, Shannon entropy and fuzzy entropy. For each industry s and time t, a short window of past returns $r_{s,t-L_{E}+1},\ldots ,r_{s,t}$ is considered. To define Shannon entropy, the returns within the window are discretized into bins or mapped to an empirical distribution with probabilities $p_{s,t,k}$ over a finite set of states k=1,…,K. The Shannon Entropy is then

(8) $$H_{s,t}=-\sum_{k=1}^{K}p_{s,t,k}\;log\;p_{s,t,k},$$

which attains higher values when the local return distribution is more dispersed and less predictable.

In the empirical implementation, Shannon entropy is computed over a rolling window of 20 trading days using a fixed number of bins K=10. Within each window, daily returns are discretized into equally spaced histogram bins spanning the range of observed returns in that window, and the empirical probabilities $p_{s,t,k}$ are obtained from the normalized histogram counts. Bins with zero probability are excluded from the summation. The natural logarithm is used, so entropy is measured in nats. This construction yields a stable, scale-consistent measure of local return dispersion that captures short-horizon uncertainty while remaining fully reproducible. Extreme forward returns are clipped at ±80% prior to target construction to protect against data errors and level shifts, but no additional winsorization is applied to the returns used in entropy estimation beyond the rolling-window restriction.

Fuzzy entropy is computed on a discrete-time series of daily returns by embedding the return series into delay vectors of dimension m and evaluating the similarity between vectors using a continuous-valued fuzzy membership function. Let

(9) $$u_{i}=\left(r_{s,t-L_{E}+i},\ldots ,r_{s,t-L_{E}+i+m-1}\right),\;i=1,\ldots ,N,$$

denote embedded vectors within a rolling window of daily observations (with sampling step Δt=1 trading day) and let $d_{ij}$ be the distance between $u_{i}$ and $u_{j}$. A fuzzy similarity is defined as

(10) $$\mu_{ij}=\exp\left(-{\left(\frac{d_{ij}}{r}\right)}^{n}\right),$$

with parameters r>0 and n>0. Fuzzy entropy quantifies the rate at which these average similarities decay as the embedding dimension increases, thereby capturing the temporal irregularity and structural complexity in the discrete return dynamics. In practice, the resulting fuzzy entropy statistic is used as a scalar feature and as a regime indicator that can modulate the influence of regression forecasts in highly irregular environments. The fuzzy entropy parameters are set to standard values commonly used in the financial time-series literature and are held fixed across horizons and model variants to ensure comparability and to avoid additional tuning degrees of freedom.

The outputs of the XGBoost models are combined into a unified predictive signal through an adaptive ensemble. Let ${\hat{p}}_{s,t}^{\left(H\right)}$ and ${\hat{R}}_{s,t}^{\left(H\right)}$ denote the XGBoost classification and regression outputs.

To combine regression outputs, predicted returns are standardized within each validation window via

(11) $$Z\left({\hat{R}}_{s,t}^{\left(H\right)}\right)=\frac{{\hat{R}}_{s,t}^{\left(H\right)}-{\bar{\hat{R}}}^{\left(H\right)}}{\sigma_{\hat{R}}^{\left(H\right)}},$$

where bars denote validation-window means and σ denotes validation-window standard deviations.

The final scalar decision score used for portfolio construction combines the probability and regression score components,

(12) $$S_{s,t}^{\left(H\right)}=w_{\mathrm{cls},H}\;{\hat{p}}_{s,t}^{\left(H\right)}+w_{\mathrm{reg},H}\;Z\left({\hat{R}}_{s,t}^{\left(H\right)}\right),$$

where $w_{\mathrm{cls},H},w_{\mathrm{reg},H}\geq 0$ and $w_{\mathrm{cls},H}+w_{\mathrm{reg},H}=1$. These weights are updated in each rolling window based on the relative out-of-sample performance of the classification and regression models, so that the decision rule places greater emphasis on the more reliable component.

Portfolio construction proceeds in two conceptually distinct stages, a maximum expected profit allocation and a minimum risk allocation. At each decision date t for a given horizon H, the set of eligible industry is defined by combining directional and magnitude criteria,

(13) $$\varepsilon_{t}^{\left(H\right)}=\left\{s\;:\;{\hat{p}}_{s,t}^{\left(H\right)}\geq \tau_{H}\;\mathrm{and}\;{\hat{R}}_{s,t}^{\left(H\right)}>0\right\},$$

where $\tau_{H}$ is the classification threshold optimized on the validation data. For industries in $\varepsilon_{t}^{\left(H\right)}$, a non-negative raw score is constructed as

(14) $$q_{s,t}^{\left(H\right)}=max\{S_{s,t}^{\left(H\right)},0\},$$

and the maximum profit portfolio assigns weights proportional to these scores,

(15) $$w_{s,t}^{\max,(H)}=\frac{q_{s,t}^{\left(H\right)}}{\sum_{j\in \varepsilon_{t}^{\left(H\right)}}q_{j,t}^{\left(H\right)}},\;s\in \varepsilon_{t}^{\left(H\right)},$$

with $w_{s,t}^{\max,(H)}=0$ for industries outside the eligible set. This allocation concentrates capital in industries with the strongest combined probability and return signals.

The minimum risk allocation is constructed from the same eligible set $\varepsilon_{t}^{\left(H\right)}$, using the estimated covariance matrix of industry returns over a trailing window. Let $\Sigma_{t}^{\left(H\right)}$ denote the covariance matrix of horizon H returns for industries in $\varepsilon_{t}^{\left(H\right)}$, estimated from historical data preceding time t. The classical long only minimum variance portfolio is obtained by solving

(16) $$\underset{w}{min}\;w^{⊤}\Sigma_{t}^{\left(H\right)}w\mathrm{subject}\;\mathrm{to}\;w^{⊤}1=1,\;w\geq 0,$$

where $w\in R^{|\varepsilon_{t}^{\left(H\right)}|}$ is the vector of industry weights and 1 is a vector of ones. Solving this constrained quadratic program exactly at each decision date would require numerical optimization. Ignoring the non-negativity constraint, the unconstrained minimum variance solution has the closed form

(17) $${\tilde{w}}_{t}^{\min,(H)}=\frac{\Sigma_{t}^{{\left(H\right)}^{-1}}1}{1^{⊤}\Sigma_{t}^{{\left(H\right)}^{-1}}1}.$$

In practice, we adopt a computationally simple approximation to the fully constrained long-only solution. Negative components of the unconstrained weight vector, if any, are truncated at zero and the remaining weights are renormalized to sum to one. This yields a feasible long-only allocation,

(18) $$w_{s,t}^{\min,(H)}=\frac{max\{{\tilde{w}}_{s,t}^{\min,(H)},0\}}{\sum_{j\in \varepsilon_{t}^{\left(H\right)}}max\{{\tilde{w}}_{j,t}^{\min,(H)},0\}}.$$

We emphasize that this truncation and renormalization step is an approximation and does not, in general, satisfy the exact optimality conditions of the constrained minimum-variance problem. Its role is to provide a stable and interpretable low-risk benchmark that can be applied consistently across rolling decision windows and model variants.

For each horizon H, both the maximum-profit portfolio ${\left\{w_{s,t}^{\max,\left(H\right)}\right\}}_{s}$ and the minimum risk portfolio $\left\{w_{s,t}^{\min,(H)}{\}}_{s}\right.$ are implemented as non-overlapping strategies, at each decision date, the portfolio weights are set based on features and model outputs up to time t, and the resulting portfolio is held unchanged for H trading days until the next decision date. This non-overlapping design ensures that holding periods do not mechanically overlap across decisions, thereby eliminating dependence arising solely from overlapping return observations. While this does not imply strict stochastic independence, since financial returns may still exhibit temporal dependence due to autocorrelation, volatility clustering, and common risk factors, it provides a cleaner and more interpretable basis for evaluating cumulative wealth paths, annualized returns, volatility, and other performance metrics in a manner consistent with the underlying forecasting horizon.

The modeling framework therefore yields three predictive architectures for each horizon. The baseline configuration excludes entropy measures, the entropy-augmented configuration incorporates Shannon entropy, and the fuzzy-entropy-augmented configuration incorporates the fuzzy-entropy statistics. Each version produces both maximum-profit and minimum-risk portfolios, resulting in a structured set of strategies that permits direct empirical comparison of the incremental value of entropy-based complexity measures. In the analysis that follows, we evaluate these models in terms of directional accuracy, relative and absolute prediction errors, cross-industry hit rates, and realized risk-adjusted returns, thereby quantifying the contribution that information-theoretic complexity features make to industry-level forecasting and allocation performance.

The choice of XGBoost as the core predictive engine is deliberate. Gradient-boosted decision trees remain a strong and widely used benchmark in financial forecasting, particularly in settings with moderate sample sizes, heterogeneous features, and strict causality constraints. Our objective is not to optimize predictive accuracy across all possible model classes, but to evaluate the incremental contribution of entropy-based complexity measures within a transparent, stable, and interpretable learning framework. Using a well-established model allows the impact of entropy features to be assessed cleanly, without confounding architectural effects. More complex deep learning architectures, such as recurrent or transformer-based models, are a natural extension of this work; however, their inclusion would shift the focus from feature-level contribution and causal evaluation to model comparison, which lies outside the scope of the present study.

## 5. Results

The empirical analysis evaluates the forecasting and portfolio construction framework across weekly, monthly, and quarterly investment horizons using both maximum-profit and minimum-risk allocation rules. The goal is not only to assess directional or statistical accuracy, but to determine how predictive signals translate into realized wealth accumulation, risk-adjusted performance, and stability across economic regimes. Because the modeling architecture produces a family of specifications, the baseline XGBoost model, an entropy augmented variant, and a fuzzy entropy augmented variant, the results quantify both the absolute performance of each strategy and the incremental economic value of incorporating information theoretic complexity measures. The analysis proceeds by examining cumulative portfolio growth, average annual returns, downside risk, and year-by-year performance dispersion, thereby providing a comprehensive picture of how each modeling choice affects investment outcomes.

All performance results reported in this section are generated strictly out of sample using the rolling-window and non-overlapping evaluation design described in Section 4. At each decision date, model estimation and portfolio construction rely exclusively on information available up to that point in time, and realized returns are computed only over the subsequent holding period. The cumulative return trajectories therefore reflect the compounding of sequential out-of-sample investment decisions over long horizons, rather than in-sample fitting. Performance is reported gross of transaction costs and market frictions. This choice is intentional and reflects the objective of isolating the incremental predictive and economic contribution of entropy-based complexity measures within a controlled setting; incorporating transaction costs would primarily affect the level of returns but would not alter the relative performance comparisons across model variants that form the central focus of the analysis.

### 5.1. Portfolio Performance Across Horizons

The results reveal a strong dependence of performance on both the investment horizon and the allocation objective. The weekly maximum-profit strategy produces the highest cumulative returns in the entire study, with the entropy-augmented model achieving more than 30,000 percent total return and an annualized return above 20 percent. The fuzzy-entropy model performs slightly below the entropy version but still substantially exceeds the baseline XGBoost. These very large cumulative gains reflect the high decision frequency and the strong presence of short-term, exploitable patterns in industry rotation, which the entropy features appear to amplify by helping the model discriminate between stable and unstable short-term return regimes.

On the monthly horizon, cumulative returns are naturally lower due to less frequent rebalancing, yet the ordering across models remains broadly consistent. The fuzzy-entropy specification produces the highest cumulative return, followed closely by the entropy model, with the baseline trailing both. Annualized returns for the three variants remain tightly clustered around 14 to 15 percent, but the entropy-based models exhibit noticeably lower annual volatility, suggesting a smoothing effect attributable to the complexity measures.

Quarterly maximum-profit strategies deliver more moderate but still impressive cumulative gains. The fuzzy-entropy model again dominates the baseline XGBoost, and entropy augmentation improves both cumulative and annualized returns relative to the baseline. The convergence of performance at this horizon suggests that long-term industrial trends are less sensitive to short-run irregularity, but that fuzzy entropy retains incremental value by describing medium-frequency nonlinear dynamics.

The minimum-risk portfolios exhibit a different performance profile. While cumulative returns fall relative to the maximum-profit portfolios, the reduction in annual return volatility is substantial, especially at the quarterly horizon where volatility drops near eleven percent. Across all horizons, the entropy-augmented and fuzzy-entropy-augmented minimum-risk strategies achieve higher cumulative returns and comparable or lower volatility than the baseline model. These improvements illustrate that entropy-based measures can enhance not only opportunity detection but also downside risk control by stabilizing the predictive inputs feeding the covariance-based allocation rule.

Table 1 demonstrates that entropy and fuzzy entropy consistently enhance long-run wealth accumulation across horizons and objectives. The gains are most pronounced in the weekly and quarterly horizons, suggesting that complexity features capture both high-frequency irregularity and medium-frequency structural variation in industry returns. 

### 5.2. Risk-Adjusted Performance, Tail Behavior, and Consistency Across Years

The Sharpe ratios summarized in Table 2, reveal several important effects of entropy augmentation. At the weekly maximum-profit horizon, the entropy model attains the highest risk-adjusted performance, with a ratio slightly above one, followed closely by the fuzzy-entropy and baseline models. This ordering aligns with the patterns observed in cumulative returns and suggests that entropy features help the model avoid extreme negative periods without sacrificing return intensity. A similar pattern emerges on the monthly horizon, although here the fuzzy-entropy model achieves the highest risk-adjusted outcome despite having slightly lower annualized returns, reflecting substantially lower volatility.

At the quarterly horizon, the fuzzy-entropy and entropy models again provide the strongest Sharpe ratios among the maximum-profit strategies. The improvement relative to the baseline reflects milder drawdowns, as evidenced by the worst-year return statistics. Both entropy-based models exhibit smaller worst-year losses than the baseline, indicating that complexity features help the model remain conservative in years with adverse macroeconomic conditions or heightened market uncertainty.

The minimum-risk strategies provide an even clearer picture. Across all horizons, entropy and fuzzy entropy improve or match the baseline Sharpe ratios, and in many cases substantially reduce worst-year losses. For instance, at the quarterly horizon, the entropy-based minimum-risk model reduces the worst-year drawdown from −17.7 percent (baseline) to −15.1 percent, while simultaneously raising the Sharpe ratio from 0.73 to 0.88. The positive-year share also increases, demonstrating greater consistency and robustness. These improvements confirm that complexity aware features enhance the stability of industry allocation decisions even when the objective is explicitly risk-minimizing.

Taken together, the Sharpe metrics and annual performance dispersion show that the inclusion of entropy and fuzzy entropy improves not only return generation but also the predictability and resilience of portfolio outcomes. Entropy-based features reduce exposure to extreme losses, increase the proportion of profitable years, and provide more balanced performance across market regimes. These findings reinforce the interpretation that information theoretic measures contribute valuable structure to the modeling of industry level return dynamics, particularly in environments characterized by regime shifts or irregular volatility patterns.

While the tabulated performance metrics provide a concise summary of long-run behavior, they necessarily compress substantial variation over time. Annual returns, regime shifts, and compounding dynamics are better understood through the visual evolution of returns across different market environments. To complement the aggregate evidence presented above, the next two sets of figures examine the temporal structure of performance. The first focuses on cross-year behavior, highlighting how each model reacts to changing volatility and industry-rotation regimes. The second documents the cumulative growth paths of all eighteen strategies, making explicit the compounding effects and drawdown patterns that underline the long-horizon results.

### 5.3. Annual Return Dynamics Across Horizons and Portfolio Types

**Figure 2:**
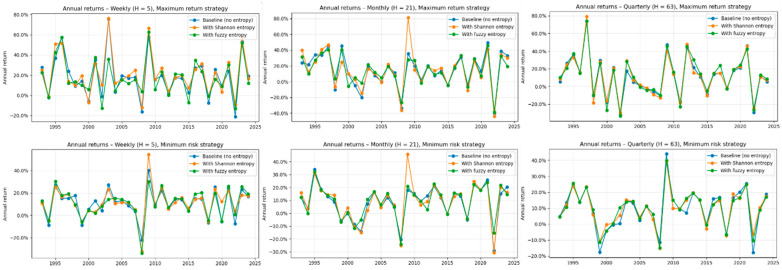
Annual industry-rotation returns for baseline, Shannon-entropy, and fuzzy-entropy models across horizons and portfolio objectives. **Notes:** The blue line corresponds to the baseline XGBoost model without entropy-based features. The orange line represents the XGBoost model augmented with Shannon entropy, and the green line represents the XGBoost model augmented with fuzzy entropy. Each panel reports year-by-year realized returns for one combination of investment horizon and portfolio objective. The top row displays results for the maximum-profit strategies at the weekly (**left**), monthly (**center**), and quarterly (**right**) horizons. The bottom row shows the corresponding minimum risk strategies. Within each panel, the blue line reports the baseline XGBoost model without complexity measures, the orange line shows performance when Shannon entropy is included as an additional feature, and the green line shows the fuzzy-entropy–augmented version. Returns are expressed as annual percentage gains or losses. The figure allows a direct visual comparison of relative stability, peak-year performance, and drawdown sensitivity across horizons and across the three modeling configurations.

The six annual return panels shown if Figure 2, are organized to isolate two central dimensions of analysis, the forecasting horizon and the portfolio construction objective. By arranging the maximum-profit strategies in the top row and the minimum-risk strategies in the bottom row, the figure highlights how the same forecasting engine behaves when allocation rules shift from aggressive exploitation of predicted return differentials to explicit minimization of portfolio variance. Horizontally, the progression from weekly to monthly to quarterly horizons reveals how the temporal scale of prediction influences volatility, responsiveness to market cycles, and the degree to which entropy-based complexity measures reshape model behavior.

Several patterns emerge from this arrangement. First, the weekly horizon exhibits the greatest visual divergence among the three models, reflecting the inherently higher noise level and stronger regime shifts at short horizons. In these panels, the entropy-augmented models, particularly Shannon entropy, tend to reduce the magnitude of extreme negative years while enhancing performance in strong positive years, generating a more symmetric profile of annual returns.

Second, the monthly horizon shows broadly similar directional movements across the three models, yet subtle differences appear during turbulent periods such as the dot-com collapse, the 2008 financial crisis, and the COVID-related regime shifts. The entropy features act as stabilizers, tempering large swings that the baseline model fails to anticipate.

Third, the quarterly horizon produces the most tightly clustered trajectories. This reflects the fact that longer horizon forecasts incorporate more historical information and thus attenuate the relative impact of short window entropy estimates. Even so, both Shannon and Fuzzy entropy continue to smooth the return path and reduce exposure to the largest drawdowns, demonstrating that local complexity measures retain predictive value even when embedded within broader forecasting windows.

Across all six panels, the arrangement of results makes explicit that the influence of entropy features is horizon dependent and interacts meaningfully with the portfolio objective. Maximum profit strategies tend to amplify beneficial effects, showing larger gains in profitable years and more moderate declines in adverse years, whereas minimum risk strategies display improvements primarily through stabilization rather than directional enhancement. The collective visual evidence confirms that information theoretic features contribute not by reshaping broad trends but by modifying the dynamics of risk, improving resilience in turbulent regimes while preserving responsiveness to industry rotation cycles.

### 5.4. Cumulative Portfolio Growth and Long-Horizon Performance

**Figure 3:**
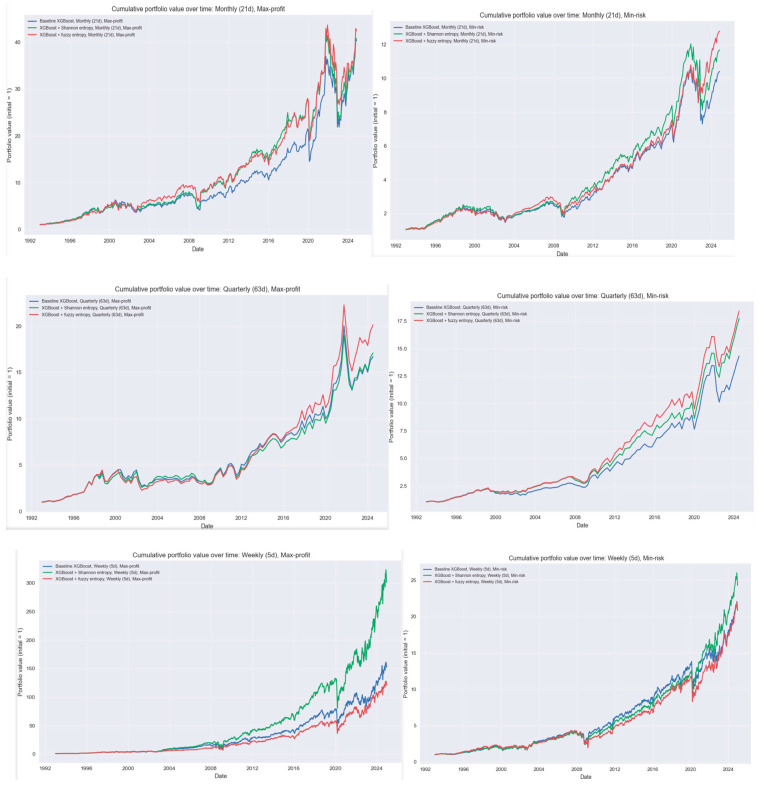
Cumulative portfolio performance for baseline, Shannon-entropy, and fuzzy-entropy models across horizons and portfolio objectives. **Notes:** The blue line corresponds to the baseline XGBoost model without entropy-based features. The green line represents the XGBoost model augmented with Shannon entropy, and the red line represents the XGBoost model augmented with fuzzy entropy. Each panel reports the cumulative wealth trajectory of a 1-dollar initial investment under one of six strategies. The top row shows the maximum profit portfolios at the weekly (**left**), monthly (**center**), and quarterly (**right**) horizons. The bottom row displays the corresponding minimum-risk strategies. The blue line corresponds to the baseline XGBoost model, the green line to the Shannon entropy augmented version, and the red line to the fuzzy entropy augmented version. The figure illustrates the long run compounding effects of model improvements, demonstrating how small differences in annual performance accumulate into substantial divergences in terminal wealth.

The cumulative return panels provide a complementary perspective to the annual return analysis, making it possible to evaluate not only the volatility and year to year behavior of each model but also the long run economic value of the forecast improvements. The organization of the panel’s mirrors that of the annual return figure, with the top row representing maximum profit allocations and the bottom row presenting minimum risk allocations. This structure permits a direct comparison of how entropy-based complexity features influence long horizon compounding under both aggressive and conservative portfolio objectives (Figure 3).

Across all horizons in the maximum profit strategies, the cumulative paths reveal three recurring themes. First, the Shannon entropy model consistently lifts the cumulative return profile, most dramatically at the weekly horizon where local return irregularities are most pronounced. Second, the fuzzy entropy model often achieves the most stable compounding during extended market cycles, particularly in the mid-2000s and post-2015 period, although it does not always dominate Shannon entropy over the long run. Third, the baseline model tends to lag over multi decade horizons even when its annual returns appear superficially similar in some periods. This divergence highlights the disproportionate importance of avoiding large drawdowns, which entropy augmented models accomplish more effectively, thereby preserving capital for reinvestment in subsequent cycles.

For the minimum risk strategies, the patterns are subtler but no less important. Entropy based models generate smoother cumulative trajectories with fewer abrupt drops, especially during crisis periods such as 1998, 2001–2002, 2008–2009, and the early 2020 COVID shock. Although the absolute level of cumulative growth is lower than in the maximum-profit counterparts by design, the entropy-enhanced models once again tend to outperform the baseline in terms of terminal wealth, confirming that the reduction in regime induced volatility translates into meaningful long horizon financial gains. Notably, the cumulative paths of the Shannon and fuzzy entropy models remain tightly clustered at the quarterly horizon, reflecting the diminished marginal value of short window complexity measures when the investment horizon already aggregates substantial temporal information.

Taken together, these six panels demonstrate that entropy informed features improve long run performance primarily through risk modulation rather than directional amplification. The mechanisms identified earlier, mitigation of extreme negative years, improved adaptation to abrupt regime changes, and smoother cross cycle adjustments, manifest clearly in compounding terms. The cumulative figures thus provide the strongest evidence that complexity-aware forecasting materially enhances the economic value of industry rotation strategies, particularly when decision frequencies are high or market regimes are unstable.

### 5.5. Comparison of Average Annual Returns

A comparison of average annual returns across horizons and portfolio objectives further clarifies the incremental contribution of entropy-based complexity features. Table 3 reports, for each horizon objective pair, the mean annual return of the baseline XGBoost model, the Shannon entropy augmented version, and the fuzzy entropy version, together with their differences relative to the baseline. Several consistent patterns emerge from this aggregation. Shannon entropy produces positive improvements across nearly all settings, with gains ranging from modest enhancements at the quarterly horizon (approximately 0.1–0.6 percentage points) to more economically meaningful increases at the weekly horizon, where the average annual return of the maximum-profit strategy rises by more than two percentage points relative to the baseline. Fuzzy entropy exhibits a different profile, outperforming the baseline in most minimum risk configurations, at both the monthly and quarterly horizons, but showing more variable behavior in maximum profit settings. These differences reflect the distinct sensitivity of the fuzzy measure to fine scale temporal irregularity, its advantages materialize most clearly in conservative allocations, where stability in local return structure directly supports risk minimizing objectives.

Taken together, the aggregated results reinforce the conclusions drawn from the annual return and cumulative return analyses. Entropy-based complexity measures systematically enhance forecasting performance and long run portfolio outcomes, but their benefits manifest differently depending on the investment horizon and the nature of the allocation rule. Shannon entropy tends to improve high frequency, high volatility strategies by moderating exposure to unstable regimes, whereas fuzzy entropy contributes more consistently to risk controlled allocations where irregularity dampening is directly rewarded. These findings provide a coherent economic interpretation of the mechanisms through which complexity aware predictors generate value and set the stage for a deeper analysis of how these models express their risk and return preferences through industry level portfolio composition.

### 5.6. Industry Allocation Patterns Across Models and Horizons

The portfolio construction framework produces distinct industry allocations depending on the forecasting horizon, the objective function, and the entropy specification. Table 4 and Table 5 report the long-run average industry weights for all strategies, offering a comprehensive view of how the baseline XGBoost model and its Shannon-entropy and fuzzy-entropy extensions allocate capital across U.S. equity industry groups. Across all horizons and model variants, the resulting portfolios exhibit a balanced and economically coherent degree of diversification. Although profit-oriented strategies naturally tilt toward industries with stronger predictive signals, the average allocations indicate that capital is distributed across a broad range of industries rather than persistently concentrated in a small subset. The effective number of industries held varies systematically with horizon and objective, reflecting differences in signal strength, covariance structure, and prevailing market regimes. Importantly, industry weights rotate over time and across strategies, with no single industry dominating allocations across horizons or entropy specifications, highlighting the adaptive and robust nature of the allocation mechanism.

Across all model configurations, REITs exhibit a remarkably stable and elevated weight under both maximum profit and minimum risk objectives, confirming their role as a persistent diversifier and a beneficiary of mean reversion dynamics at industry level. The utilities industry is similarly prominent, particularly under minimum risk allocations, where its defensive characteristics lead to all three model types assigning substantial weight in every horizon. The strong presence of utilities and real estate in risk minimizing portfolios is fully consistent with their historically low volatility and low correlation with cyclical industries and reflects the covariance driven structure of the minimum variance solution.

Cyclical growth industries such as information technology, consumer services and retail, and software tend to dominate the maximum profit allocations, especially at short horizons. This is especially visible in the weekly strategies, where momentum driven patterns are strongest and entropy extended models often assign higher weights to technology sensitive sub industry. The energy industry also appears systematically across maximum profit portfolios, reflecting its high return dispersion and the ability of tree-based models to exploit episodic trend persistence in commodity related industries.

Entropy and fuzzy entropy extensions modify these allocations in subtle but economically meaningful ways. At the monthly and quarterly horizons, both entropy specifications slightly increase exposures to stable defensive industries, such as utilities, medical industries, and pharmaceuticals, while redistributing weight away from highly volatile sub industries. This adjustment is consistent with the intended effect of entropy features, which help models recognize regimes of heightened uncertainty and shift allocations toward industries that are structurally more robust. In contrast, the weekly maximum profit strategy exhibits sharper tilts toward high beta industries, particularly information technology and specialized software or communication industries, a pattern especially pronounced in the Shannon entropy model. This feature suggests that entropy may help the algorithm identify short term episodes of strong directional predictability in growth-oriented industries, leading to amplified exposure when signal strength is high.

The minimum risk portfolios reveal a high degree of structural stability across horizons and entropy variants. Defensive industries such as utilities, real estate, insurance, banking, and diversified financials consistently receive the largest allocations, consistent with their historically low covariance structure. Entropy-based models exhibit slightly higher weights in medical and healthcare related industries, indicating that complexity measures may help identify periods in which traditional defensive industries experience elevated volatility, thereby shifting risk-minimizing allocations toward alternative stability anchors.

Taken together, the industry-allocation patterns highlight the complementary nature of technical indicators and entropy-based regime descriptors. Cyclical industries dominate when the model seeks maximum predicted return, while defensive industries anchor the minimum variance construction. Shannon entropy and fuzzy entropy modulate these allocations in ways consistent with economic intuition, entropy smooths exposures under uncertainty and helps identify stable sub industries when volatility is high, while still allowing for strong tilts toward high growth industries in periods of clear trend persistence.

## 6. Discussion and Conclusions

The empirical results provide strong evidence that incorporating information-theoretic complexity measures into industry-level forecasting models materially improves both predictive performance and portfolio outcomes. Across weekly, monthly, and quarterly horizons, the entropy-augmented XGBoost framework consistently dominates the baseline specification, demonstrating that measures of uncertainty and temporal irregularity convey economically meaningful information beyond conventional technical indicators and cross-sectional features. Because the forecasting and allocation framework is adaptive, nonlinear, and evaluated through realized portfolio outcomes rather than point forecasts, performance is assessed in economic terms through long-horizon out-of-sample returns, risk-adjusted metrics, and consistency across market regimes, rather than through parametric tests on forecast errors. Importantly, these gains are not confined to a single horizon or allocation objective, indicating that complexity-aware signals operate as a persistent and robust enhancement to machine-learning-based industry rotation strategies.

A central insight of the analysis is that Shannon entropy and fuzzy entropy capture complementary dimensions of market complexity that translate into distinct economic benefits. Shannon entropy measures the dispersion of recent return distributions and therefore reflects the degree of local uncertainty or disorder in industry dynamics. Fuzzy entropy, by contrast, captures temporal irregularity and structural instability through continuous similarity measures, allowing it to detect subtle regime shifts even when return dispersion remains moderate. When embedded as features within gradient-boosted decision trees, these entropy measures act as adaptive conditioning variables that modulate predictive reliability, particularly during periods of heightened volatility or market transition. This mechanism helps explain why entropy-augmented models deliver their largest improvements at short horizons, where informational structure evolves most rapidly.

Risk-adjusted performance metrics further clarify the nature of these gains. As shown in Table 2, the weekly maximum-profit strategy augmented with Shannon entropy achieves the highest Sharpe ratio among all configurations, reaching approximately 1.01, compared with 0.88 for the baseline XGBoost model and 0.93 for the fuzzy entropy variant. This improvement is accompanied by a markedly stronger downside profile, the worst annual return improves from −21.0% under the baseline to −11.9% with Shannon entropy, while the share of positive years increases from 81.3% to 84.4%. Crucially, this enhanced risk-adjusted performance is achieved without sacrificing upside potential, as the entropy-augmented strategy also attains the strongest best-year return of 76.2%. These results indicate that Shannon entropy improves short-horizon profitability by enhancing signal quality rather than by increasing risk exposure.

On the monthly horizon, performance differences become more nuanced but remain economically meaningful. Among maximum-profit portfolios, the fuzzy entropy specification delivers the highest Sharpe ratio at approximately 0.74, outperforming both Shannon entropy (0.62) and the baseline model (0.67), despite exhibiting a lower best-year return. This pattern suggests that fuzzy entropy contributes more to return smoothing and volatility moderation than to extreme upside capture at intermediate horizons. In minimum-risk configurations, both entropy-based models outperform the baseline, with Sharpe ratios ranging from 0.59 to 0.72 compared to 0.63 for XGBoost, alongside improved worst-year outcomes. These findings reinforce the interpretation that complexity-aware signals become increasingly valuable as horizons lengthen and portfolio objectives shift from aggressive return maximization toward stability and drawdown control.

Quarterly results further emphasize the stabilizing role of entropy-based features. In minimum-risk portfolios, both Shannon and fuzzy entropy achieve Sharpe ratios close to 0.87, substantially exceeding the baseline value of 0.73, while reducing worst annual losses to approximately −15% compared with −17.7% under the baseline. Although maximum-profit Sharpe ratios at the quarterly horizon are broadly similar across models, entropy-augmented strategies maintain competitive upside while exhibiting more balanced downside characteristics. The consistently higher proportion of positive years across entropy-based strategies, often exceeding 80% in minimum-risk configurations, underscores their ability to generate more reliable performance across market regimes.

Beyond risk-adjusted metrics, Table 1 demonstrates that the advantages of entropy-based models are also substantial in absolute economic terms. The most pronounced result again appears at the weekly horizon under the maximum-profit objective. The Shannon-entropy-augmented strategy achieves an accumulated return of approximately 30,268%, nearly doubling the total return of the baseline XGBoost strategy at 15,220% and more than doubling that of the fuzzy-entropy variant at 12,049%. This improvement is not driven by elevated volatility, as the entropy-augmented strategy simultaneously delivers a higher average annual return of 21.2% compared to 18.9% for the baseline while exhibiting slightly lower annualized volatility. These findings indicate that Shannon entropy enhances short-horizon profitability by improving predictive efficiency rather than amplifying risk.

On the monthly horizon, performance differences are more balanced but remain economically significant. Under the maximum-profit objective, all model families generate similar total returns in the range of 3900–4100%, yet entropy-based models alter the risk–return trade-off. The Shannon entropy specification achieves the highest average annual return at 15.0% but with elevated volatility, whereas the fuzzy-entropy model delivers comparable total performance with lower volatility, highlighting its stabilizing influence. In minimum-risk portfolios, both entropy-based models outperform the baseline, increasing average annual returns from 8.5% to approximately 9.1% while reducing volatility, reinforcing the role of entropy features in improving defensive allocation efficiency at intermediate horizons.

Quarterly results further illustrate the horizon-dependent benefits of complexity-aware signals. While maximum-profit strategies yield similar average annual returns across models, the fuzzy-entropy configuration achieves the highest total return at 1914% and the highest annual return at 12.3%, marginally outperforming both the baseline and Shannon entropy. In minimum-risk portfolios, entropy-based strategies again dominate, increasing average annual returns by approximately 0.6–0.7 percentage points relative to the baseline and reducing volatility from 12.9% to roughly 11.4–11.6%. These improvements translate into more resilient long-horizon allocations, where capital preservation and drawdown control are particularly important.

Industry allocation patterns further reinforce both the economic interpretability and the performance implications of the framework. Profit-oriented strategies that achieve the strongest accumulated and risk-adjusted returns, most notably the weekly maximum-profit configuration augmented with Shannon entropy, systematically concentrate capital in cyclical and growth-sensitive industries such as semiconductors, automobiles, technology hardware and equipment, banks, and energy. These allocations align closely with realized outcomes, as these strategies also exhibit Sharpe ratios above one, high best-year returns, and a large share of positive years, indicating that exposure to these industries is most effective during coherent, trend-driven market phases. On monthly and quarterly horizons, entropy-augmented profit strategies continue to favor similar growth-oriented industries but with more balanced allocations, consistent with their more moderate yet robust performance improvements.

In contrast, minimum-risk strategies that achieve superior downside protection and higher stability metrics display a markedly different industry composition. Across horizons, these portfolios allocate systematically toward utilities, food, beverage and tobacco, real estate, and consumer staples, industries associated with lower volatility and more stable cash flows. The performance outcomes closely mirror these allocations, entropy-augmented minimum-risk strategies achieve higher Sharpe ratios, smaller worst-year losses, and a greater share of positive years relative to the baseline model, particularly at monthly and quarterly horizons. Notably, the introduction of entropy-based features strengthens rather than distorts these defensive allocation patterns, producing measurable improvements in average annual returns of approximately 0.5–0.7 percentage points without increasing volatility.

From a methodological perspective, the study demonstrates how information-theoretic concepts can be operationalized within a fully causal machine-learning framework. The rolling-window design, adaptive classification thresholds, and dynamic weighting between directional probabilities and return magnitudes together form a forecasting and allocation system that is both flexible and interpretable. Rather than treating market complexity as unstructured noise, the framework explicitly quantifies it as a state variable that governs predictive reliability and allocation confidence. This approach bridges the gap between abstract measures of informational disorder and practical portfolio construction rules.

Overall, the findings indicate that market complexity is not merely a source of noise but a quantifiable and exploitable dimension of financial structure. By integrating entropy-based measures into a causal gradient-boosted framework, the proposed approach achieves superior performance, improved stability, and enhanced economic transparency. The results highlight the potential of complexity-aware machine learning systems to serve as the foundation for next-generation industry allocation strategies that adapt not only to observed price patterns but also to the evolving informational order underlying financial markets.

## Figures and Tables

**Table 1:** Total cumulative returns, average annual returns, and annual returns volatility for all model and horizon configurations.

Model Type	Horizon	Objective	Total Return	Average Annual Return	STD Annual Return
Entropy	One Week	Max Profit	30,268%	21.2%	21.0%
Fuzzy Entropy	One Week	Max Profit	12,049%	17.6%	18.9%
XGBoost	One Week	Max Profit	15,220%	18.9%	21.5%
Entropy	One Month	Max Profit	3972%	15.0%	24.1%
Fuzzy Entropy	One Month	Max Profit	4143%	14.2%	19.1%
XGBoost	One Month	Max Profit	3931%	14.5%	21.7%
Entropy	One Quarter	Max Profit	1609%	11.6%	23.4%
Fuzzy Entropy	One Quarter	Max Profit	1914%	12.3%	23.6%
XGBoost	One Quarter	Max Profit	1569%	11.5%	23.1%
Entropy	One Week	Min Risk	2334%	11.3%	13.6%
Fuzzy Entropy	One Week	Min Risk	1990%	10.8%	13.0%
XGBoost	One Week	Min Risk	1993%	10.7%	12.9%
Entropy	One Month	Min Risk	1068%	9.1%	15.5%
Fuzzy Entropy	One Month	Min Risk	1180%	9.1%	12.6%
XGBoost	One Month	Min Risk	941%	8.5%	13.6%
Entropy	One Quarter	Min Risk	1676%	10.0%	11.4%
Fuzzy Entropy	One Quarter	Min Risk	1742%	10.1%	11.6%
XGBoost	One Quarter	Min Risk	1332%	9.4%	12.9%

*Notes: “XGBoost” denotes the baseline gradient-boosted decision tree model without entropy-based features. “Entropy” and “Fuzzy Entropy” refer to otherwise identical XGBoost models augmented with Shannon entropy and fuzzy entropy features, respectively.*

**Table 2:** Sharpe ratios, best and worst annual returns, and the share of years with positive performance.

Model Type	Horizon	Objective	Sharpe Ratio	Best Year Return	Worst Year Return	Positive Year Share
Entropy	One Week	Max Profit	1.008	76.2%	−11.9%	84.4%
Fuzzy Entropy	One Week	Max Profit	0.929	62.7%	−13.0%	84.4%
XGBoost	One Week	Max Profit	0.876	75.6%	−21.0%	81.3%
Entropy	One Month	Max Profit	0.621	81.1%	−43.8%	78.1%
Fuzzy Entropy	One Month	Max Profit	0.742	45.7%	−38.6%	78.1%
XGBoost	One Month	Max Profit	0.669	49.2%	−39.8%	75.0%
Entropy	One Quarter	Max Profit	0.496	79.0%	−29.5%	68.8%
Fuzzy Entropy	One Quarter	Max Profit	0.521	73.9%	−33.2%	65.6%
XGBoost	One Quarter	Max Profit	0.498	74.3%	−32.3%	68.8%
Entropy	One Week	Min Risk	0.832	54.1%	−32.1%	87.5%
Fuzzy Entropy	One Week	Min Risk	0.829	30.3%	−33.5%	84.4%
XGBoost	One Week	Min Risk	0.831	40.1%	−21.8%	81.3%
Entropy	One Month	Min Risk	0.591	45.7%	−30.5%	78.1%
Fuzzy Entropy	One Month	Min Risk	0.716	32.2%	−24.1%	75.0%
XGBoost	One Month	Min Risk	0.626	34.0%	−29.1%	75.0%
Entropy	One Quarter	Min Risk	0.877	39.5%	−15.1%	81.3%
Fuzzy Entropy	One Quarter	Min Risk	0.872	39.8%	−15.0%	81.3%
XGBoost	One Quarter	Min Risk	0.730	44.0%	−17.7%	78.1%

*Notes: “XGBoost” denotes the baseline gradient-boosted decision tree model without entropy-based features. “Entropy” and “Fuzzy Entropy” refer to otherwise identical XGBoost models augmented with Shannon entropy and fuzzy entropy features, respectively.*

**Table 3:** A comparison of average annual returns between the XGBoost model and the entropy embedded models.

Horizon	Objective	XGBoost	Entropy	Fuzzy	Entropy Minus XGBoost	Fuzzy Minus XGBoost
One Month	Maximum Profit	14.53%	14.95%	14.17%	0.42%	−0.36%
One Month	Minimum Risk	8.50%	9.14%	9.06%	0.63%	0.55%
One Quarter	Maximum Profit	11.51%	11.60%	12.32%	0.09%	0.81%
One Quarter	Minimum Risk	9.43%	9.99%	10.14%	0.56%	0.71%
One Week	Maximum Profit	18.86%	21.22%	17.60%	2.36%	−1.25%
One Week	Minimum Risk	10.73%	11.35%	10.81%	0.62%	0.07%

*Notes: “XGBoost” denotes the baseline gradient-boosted decision tree model without entropy-based features. “Entropy” and “Fuzzy” refer to otherwise identical XGBoost models augmented with Shannon entropy and fuzzy entropy features, respectively.*

**Table 4:** Long-run average industry weights across models, horizons, and portfolio objectives.

			The Numerical Labels (1–12) in the Table Represent the Corresponding GICS Industry Names, Which Are Provided in Full Below the Table for Clarity											
Model Type	Horizon	Objective	1	2	3	4	5	6	7	8	9	10	11	12
**XGBoost**	**1 Month**	**Max Profit**	7.6%	3.2%	2.6%	9.3%	2.9%	8.9%	3.6%	3.2%	4.9%	5.8%	6.0%	2.5%
		**Min Risk**	8.4%	11.9%	9.5%	0.9%	7.9%	1.1%	5.2%	5.9%	2.4%	3.0%	1.7%	4.3%
	**1 Quarter**	**Max Profit**	8.0%	2.9%	2.1%	9.1%	1.9%	8.1%	2.1%	3.7%	6.6%	3.0%	6.5%	4.0%
		**Min Risk**	9.2%	11.1%	10.8%	1.0%	7.0%	0.7%	5.5%	4.4%	2.6%	3.4%	1.5%	3.9%
	**1 Week**	**Max Profit**	5.3%	4.3%	2.8%	9.8%	2.7%	8.9%	4.2%	3.4%	6.1%	5.7%	5.9%	2.7%
		**Min Risk**	6.6%	11.1%	9.7%	1.3%	8.0%	1.4%	5.5%	5.9%	3.3%	3.6%	1.9%	4.2%
**Entropy**	**1 Month**	**Max Profit**	7.4%	3.3%	2.6%	9.2%	3.2%	9.0%	3.4%	3.4%	4.8%	5.3%	6.1%	2.6%
		**Min Risk**	8.0%	11.6%	9.6%	1.0%	8.7%	1.1%	5.6%	5.9%	2.3%	2.8%	1.7%	4.9%
	**1 Quarter**	**Max Profit**	7.4%	3.7%	2.1%	8.8%	1.8%	7.6%	2.7%	3.2%	6.4%	3.6%	6.1%	5.2%
		**Min Risk**	9.3%	11.8%	10.4%	0.8%	7.0%	0.8%	5.1%	4.6%	3.3%	3.4%	1.5%	4.0%
	**1 Week**	**Max Profit**	5.6%	4.1%	2.6%	9.9%	2.8%	8.8%	4.3%	3.2%	6.1%	6.1%	5.8%	2.9%
		**Min Risk**	7.0%	10.8%	9.4%	1.4%	7.8%	1.5%	5.4%	6.2%	3.4%	4.0%	1.8%	4.0%
**Fuzzy**	**1 Month**	**Max Profit**	7.3%	2.6%	3.0%	9.9%	2.9%	8.6%	3.9%	3.3%	5.6%	5.4%	5.6%	2.4%
		**Min Risk**	8.3%	11.6%	10.0%	1.1%	8.0%	1.0%	5.5%	5.9%	2.5%	3.1%	1.4%	4.1%
	**1 Quarter**	**Max Profit**	8.3%	2.5%	1.9%	10.0%	2.5%	8.4%	3.3%	3.7%	6.1%	3.0%	7.4%	3.5%
		**Min Risk**	9.5%	10.9%	10.9%	0.8%	7.3%	0.7%	5.8%	4.3%	2.5%	3.5%	1.5%	4.7%
	**1 Week**	**Max Profit**	5.7%	4.4%	2.8%	9.5%	2.9%	8.7%	4.1%	3.5%	5.9%	5.6%	5.8%	2.8%
		**Min Risk**	6.7%	11.0%	9.8%	1.3%	8.2%	1.5%	5.4%	6.1%	3.3%	3.7%	1.7%	4.1%

This table reports the average industry allocations for all model structures (baseline XGBoost, XGBoost with Shannon entropy, and XGBoost with fuzzy entropy) across weekly, monthly, and quarterly horizons. The results represent averages across all non-overlapping rebalancing periods. Values are expressed as percentages of total portfolio weight and reflect the systematic tilts induced by each model and portfolio objective. Industries: 1-REITs, 2-Utilities, 3-Food, Beverage and Tobacco, 4-Semiconductors, 5-Consumer Staples, 6-Automobiles, 7-Telecommunication, 8-Household and Personal Products, 9-Banks, 10-Energy, 11-Technology Hardware and Equipment, 12-Consumer Services. Notes: “XGBoost” denotes the baseline gradient-boosted decision tree model without entropy-based features. “Entropy” and “Fuzzy” refer to otherwise identical XGBoost models augmented with Shannon entropy and fuzzy entropy features, respectively.

**Table 5:** Long-run average industry weights across models, horizons, and portfolio objectives.

			The Numerical Labels (13–25) in the Table Represent the Corresponding GICS Industry Names, Which Are Provided in Full Below the Table for Clarity												
Model Type	Horizon	Objective	13	14	15	16	17	18	19	20	21	22	23	24	25
**XGBoost**	**1 Month**	**Max Profit**	5.9%	2.8%	3.7%	3.4%	2.9%	3.7%	2.2%	4.2%	3.9%	3.2%	1.6%	1.8%	0.2%
		**Min Risk**	1.6%	4.6%	3.3%	4.6%	4.6%	4.1%	4.2%	1.9%	0.7%	2.5%	3.1%	2.5%	0.1%
	**1 Quarter**	**Max Profit**	7.0%	2.3%	3.8%	1.9%	1.9%	2.6%	2.8%	4.9%	7.5%	2.4%	2.3%	2.4%	0.3%
		**Min Risk**	1.0%	3.8%	3.4%	3.9%	4.3%	3.9%	4.8%	2.1%	2.0%	3.3%	3.8%	2.7%	0.1%
	**1 Week**	**Max Profit**	5.1%	3.9%	3.0%	2.7%	2.1%	2.7%	2.1%	4.2%	4.3%	3.1%	2.1%	2.7%	0.1%
		**Min Risk**	1.6%	4.6%	3.4%	4.2%	4.1%	3.5%	3.9%	2.0%	0.8%	3.0%	3.3%	2.9%	0.1%
**Entropy**	**1 Month**	**Max Profit**	6.3%	2.3%	3.8%	3.4%	2.5%	3.6%	2.3%	4.4%	3.7%	3.5%	1.6%	2.1%	0.2%
		**Min Risk**	1.5%	4.3%	3.1%	4.5%	4.3%	3.8%	4.5%	2.0%	0.7%	2.3%	3.4%	2.4%	0.1%
	**1 Quarter**	**Max Profit**	6.8%	2.6%	3.7%	1.9%	1.5%	2.6%	2.7%	5.9%	6.7%	2.7%	1.7%	2.3%	0.3%
		**Min Risk**	1.0%	4.3%	3.1%	3.4%	4.7%	3.5%	5.2%	1.7%	1.6%	2.7%	4.5%	2.2%	0.1%
	**1 Week**	**Max Profit**	5.0%	4.1%	2.9%	2.9%	2.5%	2.8%	1.8%	3.9%	4.2%	3.0%	1.9%	2.6%	0.1%
		**Min Risk**	1.6%	4.8%	3.3%	4.2%	4.4%	3.4%	3.7%	1.9%	0.8%	3.0%	3.1%	3.0%	0.1%
**Fuzzy**	**1 Month**	**Max Profit**	5.8%	2.5%	3.8%	3.1%	3.2%	3.1%	1.7%	4.7%	4.1%	3.5%	1.7%	2.1%	0.3%
		**Min Risk**	1.6%	4.3%	3.4%	4.4%	4.3%	4.1%	4.0%	2.1%	1.0%	2.6%	3.2%	2.4%	0.1%
	**1 Quarter**	**Max Profit**	7.1%	2.3%	4.5%	1.8%	1.7%	2.6%	3.0%	4.6%	5.2%	2.6%	2.1%	1.7%	0.3%
		**Min Risk**	1.3%	3.5%	3.4%	3.9%	4.8%	3.5%	4.4%	1.5%	1.6%	2.9%	4.4%	2.3%	0.1%
	**1 Week**	**Max Profit**	5.1%	4.0%	3.2%	2.8%	2.3%	2.6%	2.1%	3.8%	4.5%	2.9%	2.0%	3.0%	0.1%
		**Min Risk**	1.5%	4.7%	3.4%	4.1%	4.1%	3.4%	3.9%	1.9%	0.9%	2.9%	3.3%	3.0%	0.1%

This table reports the average industry allocations for all model structures (baseline XGBoost, XGBoost with Shannon entropy, and XGBoost with fuzzy entropy) across weekly, monthly, and quarterly horizons. The results represent averages across all non-overlapping rebalancing periods. Values are expressed as percentages of total portfolio weight and reflect the systematic tilts induced by each model and portfolio objective. Industries: 13-Software and Services, 14-Pharmaceuticals, Biotechnology and Life Sciences, 15-Consumer Durables and Apparel, 16-Materials, 17-Health Care Equipment and Services, 18-Media and Entertainment, 19-Commercial and Professional Services, 20-Consumer Discretionary Distribution and Retail, 21-Financial Services, 22-Transportation, 23-Capital Goods, 24-Insurance, 25-Real Estate Management and Development. Notes: “XGBoost” denotes the baseline gradient-boosted decision tree model without entropy-based features. “Entropy” and “Fuzzy” refer to otherwise identical XGBoost models augmented with Shannon entropy and fuzzy entropy features, respectively.

## Data Availability

Restrictions apply to the availability of these data. Data were obtained from MSCI Institute and are available at https://www.msci.com/indexes/index-resources/gics (accessed on 13 December 2025) with the permission of MSCI Institute.
